# Comparative Study on the Formation, Structure, and Properties of Granular Cold-Water-Swelling Oat Starch by Subcritical Ethanol–Water Treatment at High and Low Temperatures

**DOI:** 10.3390/foods15193506

**Published:** 2026-10-01

**Authors:** Yue Teng, Zijie Xie, Maojun Yao, Ji Yu, Yuntong Li

**Affiliations:** Hunan Provincial Key Laboratory of Research, Resource Mining and High-Valued Utilization on Edible & Medicinal Plant, College of Chemistry and Chemical Engineering, Jishou University, Jishou 416000, China; yueteng@stu.jsu.edu.cn (Y.T.); xiezijie@stu.jsu.edu.cn (Z.X.); yaomaojun@126.com (M.Y.); sunofwind8003@outlook.com (J.Y.)

**Keywords:** subcritical ethanol–water modification, oat starch, granular cold-water-swelling starch, crystalline structure, modification temperature

## Abstract

Granular cold-water-swelling oat starch was prepared under both low-temperature (90–130 °C) and high-temperature (150–190 °C) conditions using subcritical ethanol–water treatment, and the resulting structural and property changes were systematically investigated. The low-temperature treatment largely retained the native orthorhombic crystal structure and produced granules with finer morphology, whereas the high-temperature treatment induced crystallization of a new orthorhombic amylose phase and generated a swollen granular state with superior cold-water swelling capacity (11.60 g/g vs. 1.99 g/g for native starch). X-ray diffraction analysis showed that the proportion of nanocrystals with the native orthorhombic crystal structure decreased, while the proportion of the new orthorhombic phase increased with increasing temperature. The gelatinization endotherm of modified oat starch obtained by the high-temperature method disappeared. The molecular weight of modified oat starch exhibited a broad distribution, with weight-average molecular weight (Mw) values ranging from 86,633.57 to 140,842.31 kDa and a polydispersity index (PDI) between 5.80 and 9.70. These findings demonstrate that elevated temperatures primarily drive the crystallization of the new orthorhombic amylose phase, providing a foundation for designing functional starch-based materials with tailored properties.

## 1. Introduction

Starch is widely used as a functional ingredient in the food industry owing to its swelling, gelatinization, gelling, and emulsifying properties [1]. Among starch sources, oat starch possesses a smooth particle surface, short amylose chains, high relative crystallinity, and good water solubility and swelling capacity, making it a promising candidate for food processing applications [2]. However, native starch suffers from inherent limitations, including high thermal viscosity, low solubility, instability under thermal and shear stress, rapid retrogradation, and poor refrigerated stability, which severely restrict its practical utility [3]. Physical modification technology is an economical and eco-friendly technology that can be used to modify starch properties [4]. Wet heat treatment, in particular, can effectively alter starch crystallization and gelatinization behavior.

Cold-water-swellable starch is divided into pregelatinized starch and granular cold-water-swelling (GCWS) starch. Both types can be dispersed in water at room temperature and swell rapidly into a paste, enhancing processing convenience [5]. However, powdered starches cannot be easily dispersed in cold water due to clumping [6]. GCWS starch largely retains its original granular state while absorbing water and swelling rapidly in cold water, making it suitable for gelling agents in jellies, jams, puddings, desserts, and sausages. Subcritical fluids—characterized by high density, low viscosity, and strong penetrating ability—can efficiently alter the multiscale structure of macromolecular polymers under mild conditions and have been applied in graft and blend modification of polymers [3,7]. In this context, granular starch containing single-helical amylose can be prepared: following starch gelatinization, amylose forms a single-helical structure in the presence of suitable guest molecules, and these helices subsequently assemble into an ordered crystalline phase [8]. The resulting material features hydrophobic cavities and is used as a novel resistant starch, bioactive carrier, and food improver [9].

Existing preparation methods for GCWS starch—including dual-nozzle spray drying, ethanol-alkali treatment, high-temperature/high-pressure alcohol processing, and atmospheric polyol treatment—suffer from high energy consumption, solvent residue, and product structural heterogeneity. In contrast, subcritical fluid treatment combined with ethanol-heat processing enables the safe and rapid preparation of GCWS starch [3], offering a new pathway for high-value utilization of oat starch. Both the high-temperature/high-pressure alcohol method (149–182 °C) reported by Dries [10] and the lower-temperature approach (95 °C) reported by Zhang et al. [11] can produce intact GCWS starch. The underlying principle in both cases is the controlled absorption of water to achieve optimal starch swelling while ethanol reinforces particle surface toughness. However, existing studies have focused primarily on ambient-pressure conditions, narrow temperature ranges, and limited starch concentrations. Systematic comparisons across a wide temperature range remain lacking. Specifically, it is unclear how the modification temperature affects the structure and properties of GCWS starch, and whether the excellent cold-water gelation properties obtained under high-temperature/high-pressure conditions can be achieved at reduced temperatures.

This study examined how the modification temperature and ethanol concentration govern the crystallization of a new phase in oat starch. The intrinsic nanocrystals in native oat starch have orthorhombic and/or hexagonal crystal structures, which are responsible for its structural properties. The morphology (SEM), crystalline structure (XRD), functional-group composition (FTIR), and thermal properties (DSC) were characterized before and after subcritical ethanol–water treatment, and the formation of the new, non-complex crystal structure induced by the treatment was investigated.

A note on terminology is required before the results are presented. First, starches classified as A-, B- and C-type are not polymorphs, and the corresponding crystal structures are not three polymorphs of one substance. Starches classified as A-type contain nanocrystals with an orthorhombic crystal structure, those classified as B-type contain nanocrystals with a hexagonal crystal structure, and those classified as C-type contain a mixture of orthorhombic and hexagonal nanocrystals in starch. Second, the letter V in the expression “V-type” does not designate a crystal structure. The designation originated from the diffraction signature recorded for gelatinized (pasted) starch and for amylose–guest complexes, and it has since been applied to the single-helical amylose phase. It is therefore the description of a diffraction signature, not one of the 14 Bravais lattices. In this work the new crystalline phase formed during subcritical ethanol–water treatment is named the orthorhombic ethanol-induced amylose phase (OEA phase). The name identifies the crystal system and the process that generates the phase, and it is used as a working designation pending full indexing of the structure.

On the basis of the crystallographic considerations set out above, three hypotheses were tested. H1, the phase formed at high temperature, is not a new polymorph of the native nanocrystals but a separate crystalline phase. H2, the native nanocrystals, are not converted in the solid state but are solvated, and the new phase crystallizes from the liquid phase. H3, the hydroxyl groups of ethanol, and not temperature alone, direct this crystallization. The results are presented in the order in which they bear on these hypotheses, and the Discussion develops the mechanism that is consistent with the data.

## 2. Materials and Methods

### 2.1. Materials

Oat grains were purchased from Shenyang Xinchang Grain Trading Co., Ltd. (Shenyang, Liaoning, China). Anhydrous ethanol and n-Hexane were obtained from Chengdu Kolon Chemicals Co., Ltd. (Chengdu, Sichuang, China). Neutral protease (enzyme activity: 100,000 U/g) was purchased from Shandong Lonct Enzymes Co., Ltd. (Linyi, Shandong, China). Lithium bromide was purchased from Sigma Chemical Company (St. Louis, MO, USA). Dimethyl sulfoxide (Chromatographic grade) was purchased from Shanghai Anpu Experimental Technology Co., Ltd. (Shanghai, China). Potassium bromide was purchased from Sinopharm Chemical Reagents Co., Ltd. (Shanghai, China). All other chemicals and reagents were of analytical grade. Deionized water was used throughout the experiments.

### 2.2. Preparation of Oat Starch

The oat starch was prepared according to the method described by He et al. [12] with some modifications. Oat grains were weighed, cleaned, ground in high-speed multifunctional grinder (JP-500C, Yongkang Jiupin Industry & Trade Co., Ltd., Jinhua, Zhejiang, China), and sieved through an 80-mesh screen. Whole oat flour was mixed with *n*-hexane at a liquid-to-solid ratio of 1:1 (mL/g), soaked to remove fat, and dried to obtain defatted oat flour. The defatted oat flour and water were mixed thoroughly in a 1:2 (g/g) ratio, and 2% (*w*/*w*, relative to dry flour) neutral protease (2 g enzyme per 100 g dry flour) was added. The mixture was stirred (40 °C, 10 h) under heated magnetic stirrer (LC-OB-BL, Shanghai Lichenbangxi Instrument Technology Co., Ltd., Shanghai, China). Upon completion of the reaction, the mixture was centrifuged (3880× *g*, 15 min) using a low-speed centrifuge (DL-4C, Shanghai Anting Scientific Instrument Factory, Shanghai, China); the supernatant was removed, the precipitate was washed with water, and the suspension was passed through a 300-mesh gauze. The filtrate was filtered again, the supernatant was removed, and the surface layer of the starch precipitate was scraped off. This washing process was repeated 2–3 times. The precipitate was dried (40 °C, 24 h) in an electric forced-air drying oven (BGZ-146, Shanghai Boxun Industrial Co., Ltd. Medical Equipment Factory, Shanghai, China) and ground to obtain oat starch. The moisture content of the extracted oat starch was determined to be 7.9% with a halogen moisture analyzer before the modification experiments.

### 2.3. Preparation of Subcritical Fluid-Modified Oat Starch

Subcritical fluid-modified oat starch was prepared according to the method described by Li et al. [3] with some modifications. It was mixed with ethanol concentrations (*v*/*v*, 50%, 70%) at a solid-to-liquid ratio of 1:9 (g/mL) and stirred until uniform. The mixture was transferred to high-temperature reactor (KH-50 mL, Shanghai Xiniu Laiber Instrument Co., Ltd., Shanghai, China), with different modification temperatures (90, 110, 130, 150, 170, 190 °C) held for 20 min, a time adopted from a previously reported subcritical ethanol–water protocol [3] that was sufficient for starch modification without excessive degradation. In this study, 90–130 °C was defined as the low-temperature range and 150–190 °C as the high-temperature range. The fluid therefore remained in the subcritical state. The estimated autogenous pressures ranged from ≈0.09 MPa (90 °C, 50% ethanol) to ≈1.85 MPa (190 °C, 70% ethanol) (Table S1). At 190 °C the estimated autogenous pressure is ≈1.85 MPa, which is far below the critical pressure of the ethanol–water system (ethanol 6.14 MPa; water 22.1 MPa), and the temperature is below the critical temperature (ethanol 241 °C; water 374 °C). After the mixture cooled, anhydrous ethanol (22.5 mL) was added, and the mixture was filtered using medium-speed qualitative filter paper, followed by 2–3 washes with anhydrous ethanol. The modified oat starch was dried at 40 °C overnight in oven, ground in a mortar, sealed in self-sealing bags, and stored for future use. The modified starch was designated as OS_T/C%_, where *T* represents the modification temperature and *C* represents the ethanol concentration used in the reaction.

### 2.4. Optical Microscopy

Following the method described by Guo [13] with minor modifications, 0.1 g of oat starch was weighed and mixed with a water-glycerol solution (1:1 *v*/*v*) to prepare a 2% (*w*/*v*) starch suspension. This suspension was spread onto a glass slide, and the starch particles were observed and photographed using a polarizing microscope (cx40p, Ningbo Shunyu Instruments Co., Ltd., Ningbo, Zhejiang, China) at 500× magnification under appropriate lighting conditions.

### 2.5. Scanning Electron Microscopy (SEM)

A scanning electron microscope (QUANTA 250 FEG, Thermo Fisher Scientific, Waltham, MA, USA) was used to examine the morphology and surface features of starch samples. A small amount of sample was dispersed on an aluminum tray coated with conductive double-sided tape and gold-sputtered under vacuum conditions. The samples were observed and photographed using the SEM at magnification (×5000), and the SEM images were recorded.

### 2.6. Wide-Angle X-Ray Diffraction (XRD)

The method described by Zhan et al. [14] was followed with appropriate modifications. The crystal structure of starch was analyzed using an X-ray diffractometer (D8 Advance, Bruker Technology Co., Ltd., Beijing, China). Each oat starch sample was equilibrated at room temperature for 24 h. The powdered samples were evenly distributed in the center of the circular groove on the sample holder and compacted using a glass slide. The scanning rate was set to 2°/min, and the scanning range was set between 5° and 40°.

Relative crystallinity (RC) was calculated by peak fitting in MDI Jade 6. Patterns were analysed over 2θ = 5–35°. A linear background was fitted between 5° and 35° and subtracted, and the residual profile was fitted until the fit stabilized. Reflections were constrained to the positions listed in Table S2, so that the overlapping reflections of the native orthorhombic nanocrystals and of the new orthorhombic phase were separated and the crystalline area attributed to each phase. RC was calculated as the total crystalline area divided by the total area of the pattern, RC = (area of crystalline peaks/total area) × 100%. The interplanar spacing (d) was calculated from the diffraction angle (2θ) using the Bragg equation, d = λ/(2 sin θ), with λ = 1.5406 Å (Cu Kα1).

### 2.7. Fourier Transform Infrared Spectroscopy (FT-IR)

The starch samples were measured using an FTIR (IS50, Thermo Fisher Scientific, Waltham, MA, USA), following the method described by Li et al. [15], with minor modifications. The samples were dried overnight to equilibrate moisture content. Air measurements were taken as blank backgrounds. Starch (1 mg) was mixed uniformly with KBr (100 mg) at a ratio of 1:100 (*w*/*w*), and the mixture was pressed into a pellet. Spectral scans were performed in the range of 4000–400 cm^−1^ with a resolution of 4 cm^−1^ and 16 passes. The IR spectra were automatically baseline-corrected and smoothed using OMNIC software (Version 9.0, Thermo Fisher Scientific, Waltham, MA, USA), followed by Fourier deconvolution in the range of 1200–800 cm^−1^.

### 2.8. Differential Scanning Calorimeter (DSC)

Differential scanning calorimetry (DSC) was performed using a METTLER TOLEDO TGA/DSC 3+ instrument equipped with STARe software (Version 16.40, Mettler Toledo, Greifensee, Switzerland) under a nitrogen purge gas. Samples (sample: deionized water = 1:2, *w*/*w*, dry basis) were sealed in standard 40 µL aluminum crucibles and equilibrated at room temperature for 12 h. Testing was then conducted, using an empty crucible as reference. Test conditions were as follows: the temperature range was 30–150 °C, and the heating rate was 10 °C/min. DSC was performed once per sample and is reported as a representative characterization, the thermograms were evaluated over the 40–90 °C window.

### 2.9. Gel Permeation Chromatography (GPC)

Starch (5 mg) was dissolved in 5 mL of the mobile phase (DMSO) by heating at 80 °C for 3 h, and the solution was then filtered through a 0.45 μm filter [16]. A high-performance size exclusion chromatography system (Ultimate 3000, Thermo Fisher Scientific, USA) equipped with a multiangle light scattering detector (DAWN HELEOS-II, Wyatt Technologies, Inc., Santa Barbara, CA, USA) and a refractive index detector (OPTILAB T-rex, Wyatt Technologies, Inc., Santa Barbara, CA, USA) was used to analyze the molecular weight of the starch, with 0.5% LiBr-containing DMSO as the mobile phase at a controlled temperature of 60 °C [17,18], the flow rate was 0.3 mL/min with isocratic elution over 120 min, and the injection volume was 200 μL. The chromatographic columns were Ohpak SB-805 HQ, Ohpak SB-804 HQ, and Ohpak SB-803 HQ (300 × 8 mm). Weight- and number-average molecular weights (Mw, Mn) were calculated directly from the MALLS and RI signals using the refractive index increment (dn/dc = 0.07 mL/g) for starch in DMSO/LiBr; because MALLS provides absolute molecular weights, no calibration standards were required for quantification.

### 2.10. Cold Water Swelling and Solubility Properties

Based on the method described by Gani [19], with minor modifications, the swelling measurement used gentle agitation (200 rpm, 30 min) to avoid disrupting the swollen granules, whereas the solubility measurement used vigorous stirring (2200 rpm, 3 min) to achieve complete dispersion of soluble starch. Starch (40 mg, dry basis) was dispersed in 1.0 mL of distilled water (4% *w*/*v*). Place the tube in a water bath shaker at 25 °C and maintain at 200 rpm for 30 min. Centrifuge (15,000× *g*, 3 min) with a bench-top high-speed centrifuge (TGL-15B, Shanghai Anting Scientific Instruments Co., Ltd., Shanghai, China). Calculate the cold-water swelling capacity using Equation (1), and express the result in g/g.

Mix 0.2 g of starch with 20 mL of distilled water on a magnetic stirrer at 2200 rpm for 3 min. Centrifuge the suspension at 2190× *g* for 5 min. Transfer 10 mL of the supernatant to a watch glass and dry it at 105 °C for 4 h. Calculate the value according to Equation (2).

(1) $$\mathrm{swelling}\;(g/g)=\frac{m_{1}}{m_{0}\times \left(1-\mathrm{MC}\right)}$$

(2) $$\mathrm{Solubility}\;(\%)=\frac{{2\times m}_{2}}{m_{0}\times \left(1-\mathrm{MC}\right)}\times 100\%$$

where *m*_0_ is the sample mass (g), *m*_1_ is the mass of the wet sediment (g), *m*_2_ is the mass of the dried solids from the 10 mL supernatant aliquot (g), *MC* is the moisture content (fraction), and the factor 2 accounts for sampling only 10 of 20 mL of the supernatant.

All masses are expressed on a dry-weight basis using the moisture content determined with a halogen moisture analyzer.

### 2.11. Gel Form

Add 0.5 g starch to a small glass vial containing 4.5 g of distilled water, let it stand for 10 min, and then photograph the modified oat starch gel for observation.

### 2.12. Statistical Analysis

Quantitative assays were performed as follows: XRD, cold-water swelling, and cold-water solubility, *n* = 2 independently prepared sample batches (reported as mean ± SD); DSC, FTIR, and GPC, performed once per sample and reported as a representative characterization without standard deviation. Technical repeats were not treated as independent replicates. Curves were plotted using Excel 2010 and Origin 2025b software; XRD experimental data were processed using MDI Jade 6 software; deconvolution was performed using OMNIC software. For cold-water swelling and solubility, two-way ANOVA (temperature × ethanol concentration) was performed with native oat starch handled as a prespecified control, followed by Tukey’s HSD post hoc test (SPSS 27.0, *p* < 0.05).

## 3. Results

### 3.1. Optical Microscopic Analysis

**Figure 1:**
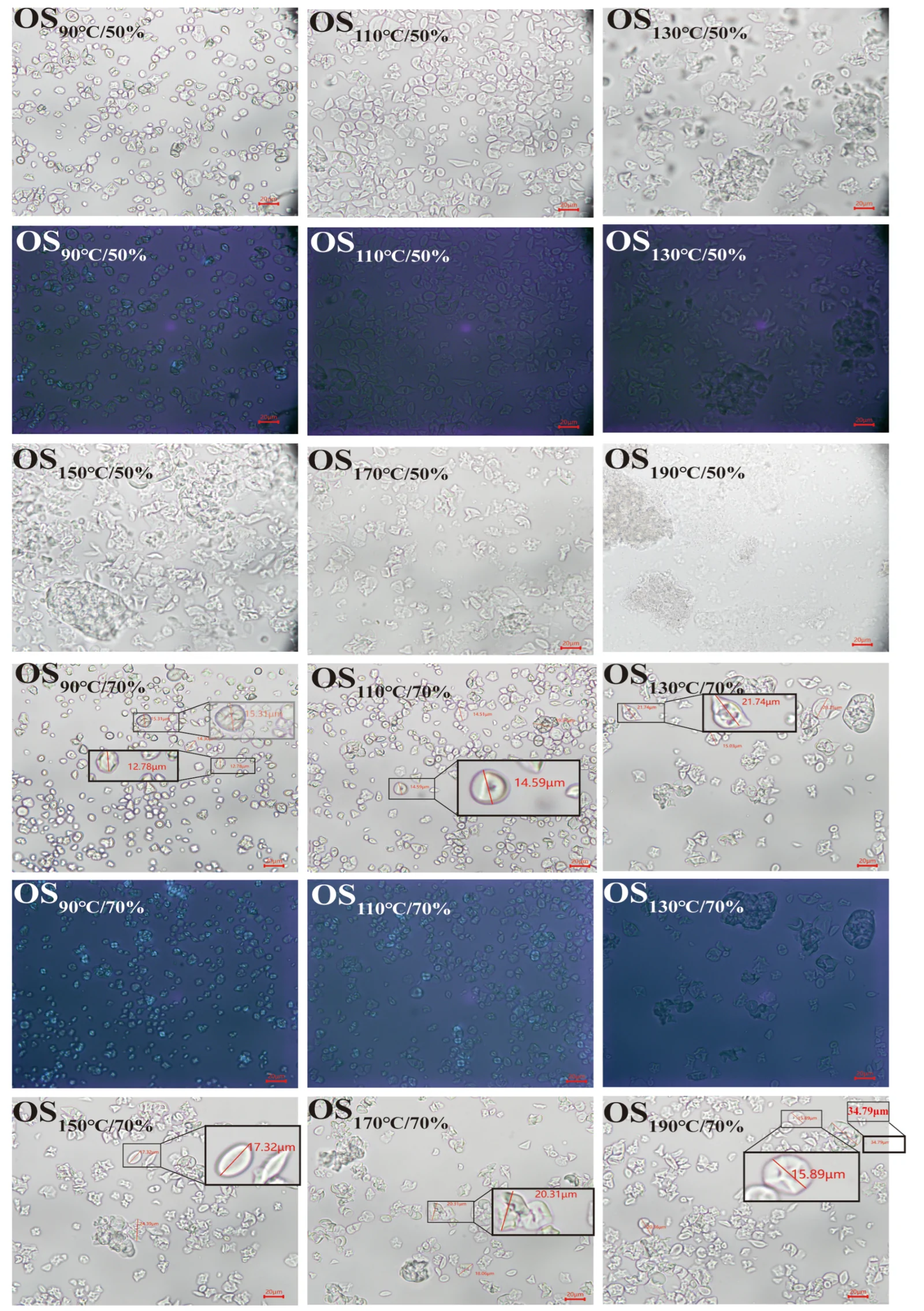
Optical micrographs (polarized light, 500×) of native and subcritical ethanol–water-treated oat starch (50% and 70% *v*/*v* ethanol, 90–190 °C), imaged in aqueous glycerol (hydrated state). The bright birefringence (Maltese cross) indicates residual crystalline order; its disappearance with increasing temperature reflects loss of granular order.

The microstructure of modified oat starch is shown in Figure 1, with the particle morphology and optical properties of native oat starch provided in the Supplementary Material (Figure S1). Native oat starch showed oval or irregular shape [20]. At 90 °C (OS90 °C/50%) and 110 °C (OS110 °C/50%), the starch granules remained largely intact. At 130 °C (OS130 °C/50%), partial clumping occurred. With further temperature increases to 150 °C (OS150 °C/50%) and 170 °C (OS170 °C/50%), the extent of swelling and distortion became evident. Ultimately, at 190 °C (OS190 °C/50%), severe caking and a gel-like consistency were observed (Figure 1), suggesting complete loss of granular structure.

The polarized light cross (Maltese cross) disappeared at 110 °C under 50% ethanol concentration (OS_110°C/50%_). When the ethanol concentration was increased from 50% to 70%, the temperature at which the polarized cross disappeared was delayed from 110 °C (OS_110°C/50%_) to 130 °C (OS_130°C/70%_).

### 3.2. Morphological Analysis

**Figure 2:**
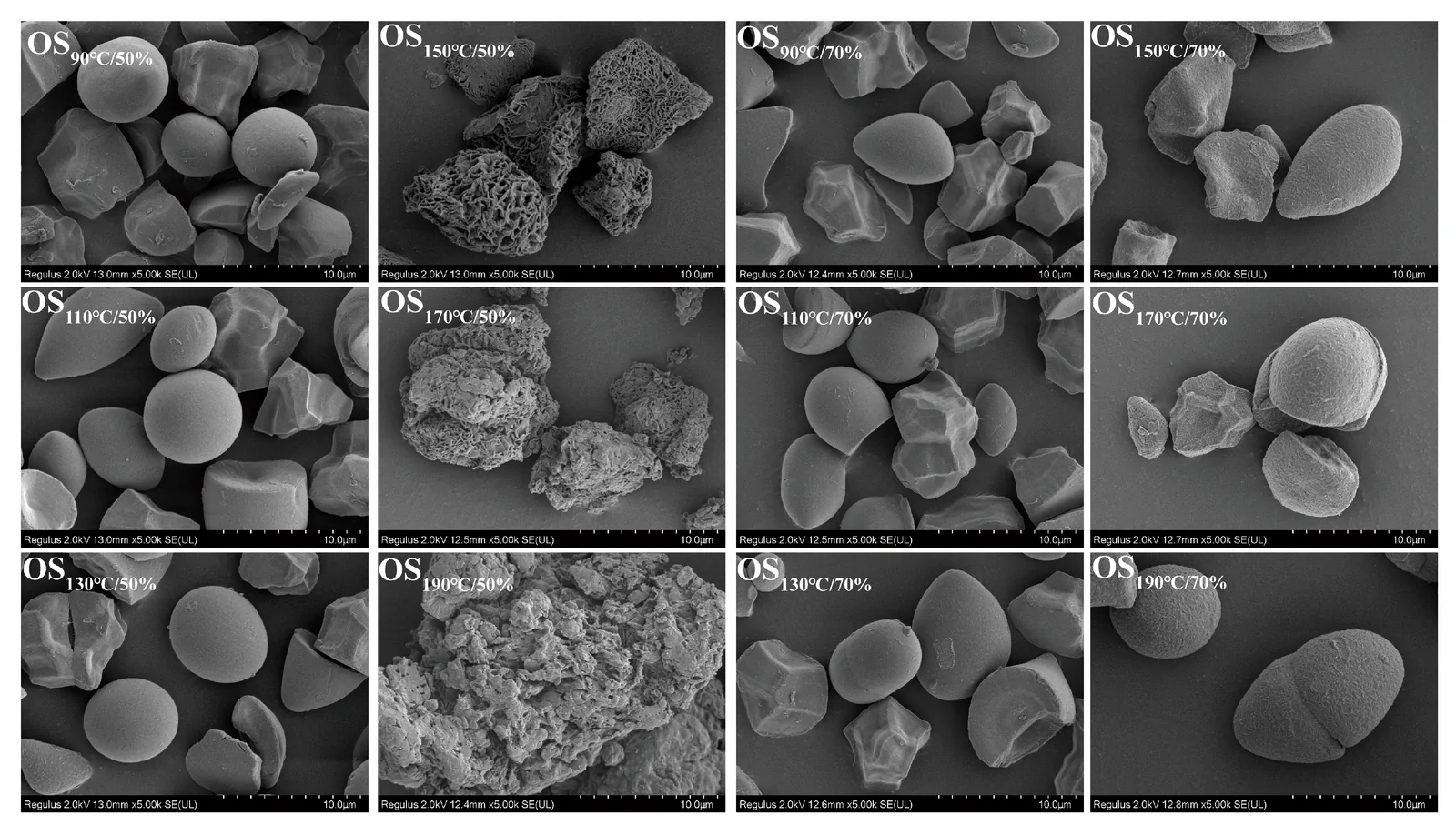
Scanning electron microscopy (SEM) images (5000×) of native and subcritical ethanol–water-treated oat starch (50% and 70% *v/v* ethanol, 90–190 °C), imaged after drying, showing granular morphology and surface structure.

Figure 2 shows scanning electron microscope (SEM) images of modified oat starch. SEM images of native oat starch and modified oat starch at various magnifications are included in the Supplementary Materials (Figure S2).

The native oat starch granules showed a polygonal and oval shape, with smooth, pore-free surfaces at high magnification (×5000). At 50% ethanol concentration, oat starch retains its particle integrity and original morphology when modification temperature ranges from 90 to 130 °C. Upon heating to 150 °C, the particles transform into a loose, porous honeycomb structure. Further heating to 170 °C, the surfaces rupture, agglomerate into clumps, resulting in severe destruction of the original structure.

Oat starch modified at 150 °C in 50% ethanol (OS_150°C/50%_) retained its granular morphology and exhibited a loose, porous honeycomb structure, whereas the sample modified at 170 °C (OS_170°C/50%_) underwent severe deformation, with aggregating into clumps, and the surface showing wrinkling and cracking. In contrast, at 70% ethanol concentration, the modified oat starch retained its expanded granular morphology even after high-temperature treatment, with only a slightly roughened surface.

### 3.3. Crystal Structure Analysis

**Figure 3:**
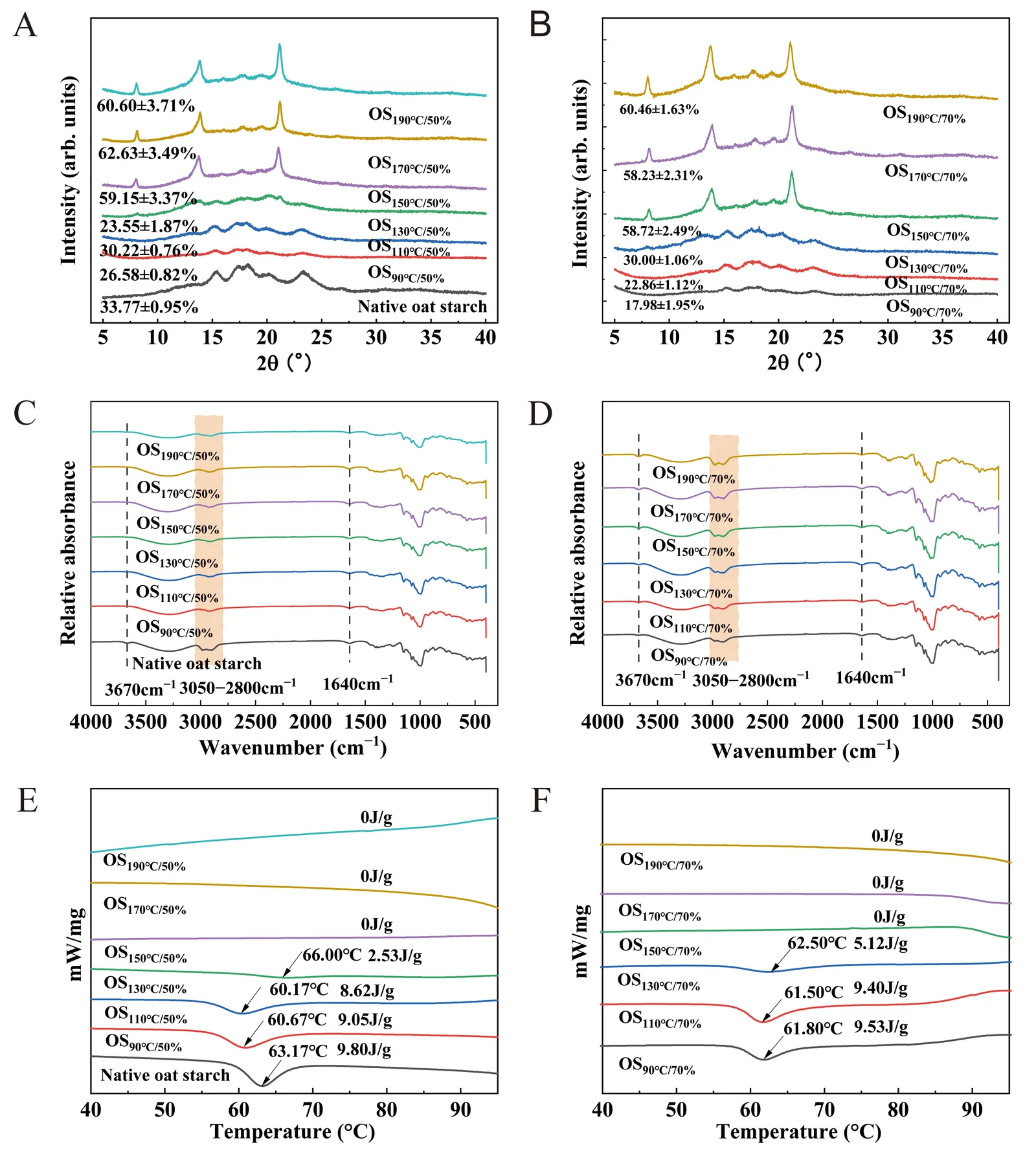
(**A**,**B**) X-ray diffraction (XRD) patterns, (**C**,**D**) infrared (IR) spectra, and (**E**,**F**) differential scanning calorimetry (DSC) thermograms of native and subcritical ethanol–water-treated oat starch prepared at 90–190 °C with 50% or 70% (*v*/*v*) ethanol.

Figure 3A,B illustrate the XRD patterns of modified oat starch. Native oat starch exhibited characteristic diffraction peaks at 2θ ≈ 15.4°, 17.3°, 18.3°, 20.0° and 23.3°, which are the reflections of the orthorhombic nanocrystals that give A-type starches their characteristic pattern. From a crystallographic perspective, starches classified as A-type contain nanocrystals with an orthorhombic crystal structure, starches classified as B-type contain nanocrystals with a hexagonal crystal structure, and starches classified as C-type contain a mixture of orthorhombic and hexagonal nanocrystals in starch [21]. These designations classify the starch and not polymorphs of a single crystal structure. As the modification temperature increased, the reflections of the native orthorhombic nanocrystals gradually weakened, while a new set of reflections appeared at 2θ ≈ 8.0°, 13.9° and 21.2° at 150 °C. These reflections cannot be indexed on the unit cell of the native nanocrystals, which indicates that a second, distinct crystalline phase is formed rather than that the native lattice is distorted. The new phase is designated the orthorhombic ethanol-induced amylose phase (OEA phase). Its three reflections are consistent with the V6a-type anhydrous orthorhombic unit cell of single-helical amylose (a ≈ 13.0 Å, b ≈ 23.0 Å, c ≈ 8.05 Å), whose characteristic reflections have been reported at 2θ ≈ 7.9°, 13.7° and 21.0° [22].

As the modification temperature continued to rise, the area of the reflections of the new phase increased progressively. At higher temperatures (150–190 °C), the modified oat starch showed a higher relative crystallinity (62.63 ± 3.49%) than native oat starch (33.77 ± 0.95%), and this net increase in crystalline order coincides with the disappearance of the gelatinization endotherm. Both observations are consistent with the solvation of the native nanocrystals and the crystallization of a new phase from the liquid phase, and they are difficult to reconcile with a transition within a single lattice. In addition, at the same modification temperature (150–190 °C) the reflections of the new phase were more intense at 50% ethanol than at 70% ethanol, indicating that the higher ethanol concentration limited the amount of amylose available for crystallization while better preserving granular integrity.

All diffraction peaks of the modified oat starch are therefore consistent with the orthorhombic crystal system. The native reflections are consistent with the orthorhombic unit cell of the A-type nanocrystals and the new reflections with a V6a-type orthorhombic unit cell of single-helical amylose, and none of the observed peaks requires the assumption of a Bravais lattice other than orthorhombic (the complete peak list is given in Table S2 of the Supplementary Materials). Given that the exact space group and solvent occupancy of the new phase have not been fully established, this interpretation is presented as being consistent with the proposed orthorhombic phase rather than as a definitive structural determination.

### 3.4. IR Spectroscopy Analysis

Figure 3C,D present the IR spectra of the modified oat starch. All samples exhibited consistent absorption bands across the spectral range, indicating that the modification temperature did not significantly alter the functional-group composition of the starch system. These observations indicate that the formation of the new crystalline phase (Section 3.3), induced by ethanol molecules, proceeds through noncovalent reorganization of the starch components (primarily amylose), without the formation of new covalent bonds [23]. Characteristic absorption bands were observed at 3670 cm^−1^, 3050–2800 cm^−1^, and 1640 cm^−1^, which are attributed to the stretching vibrations of –OH and –CH_2_– groups in the glucose units, as well as hydrogen bonding between glucose units and water molecules [24]. Due to the low concentration and nanoscale nature of the nanocrystals of the new orthorhombic phase, their characteristic IR bands cannot be unambiguously resolved, and IR is therefore used only as complementary, not direct, evidence for this phase.

### 3.5. Thermal Properties

The thermal behavior of the samples was characterized by differential scanning calorimetry (DSC). Gelatinization is an endothermic transition in which the ordered system (nanocrystals in starch), due to heat and an excess of water, undergoes an irreversible transition; the nanocrystals within starch are solvated [25,26]. The DSC curves are presented in Figure 3E,F. For native oat starch, an apparent gelatinization endothermic peak was observed at 63.17 °C (Figure 3E). As the modification temperature increased, the intensity of this gelatinization peak progressively weakened. At 150 °C, the gelatinization endotherm completely disappeared for both ethanol concentrations (Figure 3F), which was consistent with the research conducted by Shi [22]. Notably, the OS_190°C/70%_ sample retained granular morphology (Figure 2) and exhibited pronounced crystallinity (Figure 3B). However, its gelatinization endothermic peak completely disappeared on the DSC thermogram (Figure 3F).

The loss of the gelatinization endotherm while the total crystallinity of the sample increases is a further indication that the native nanocrystals are solvated and that a new crystalline phase, rather than a new polymorph of the native nanocrystals, is formed.

### 3.6. Molecular Weight of Starch

**Figure 4:**
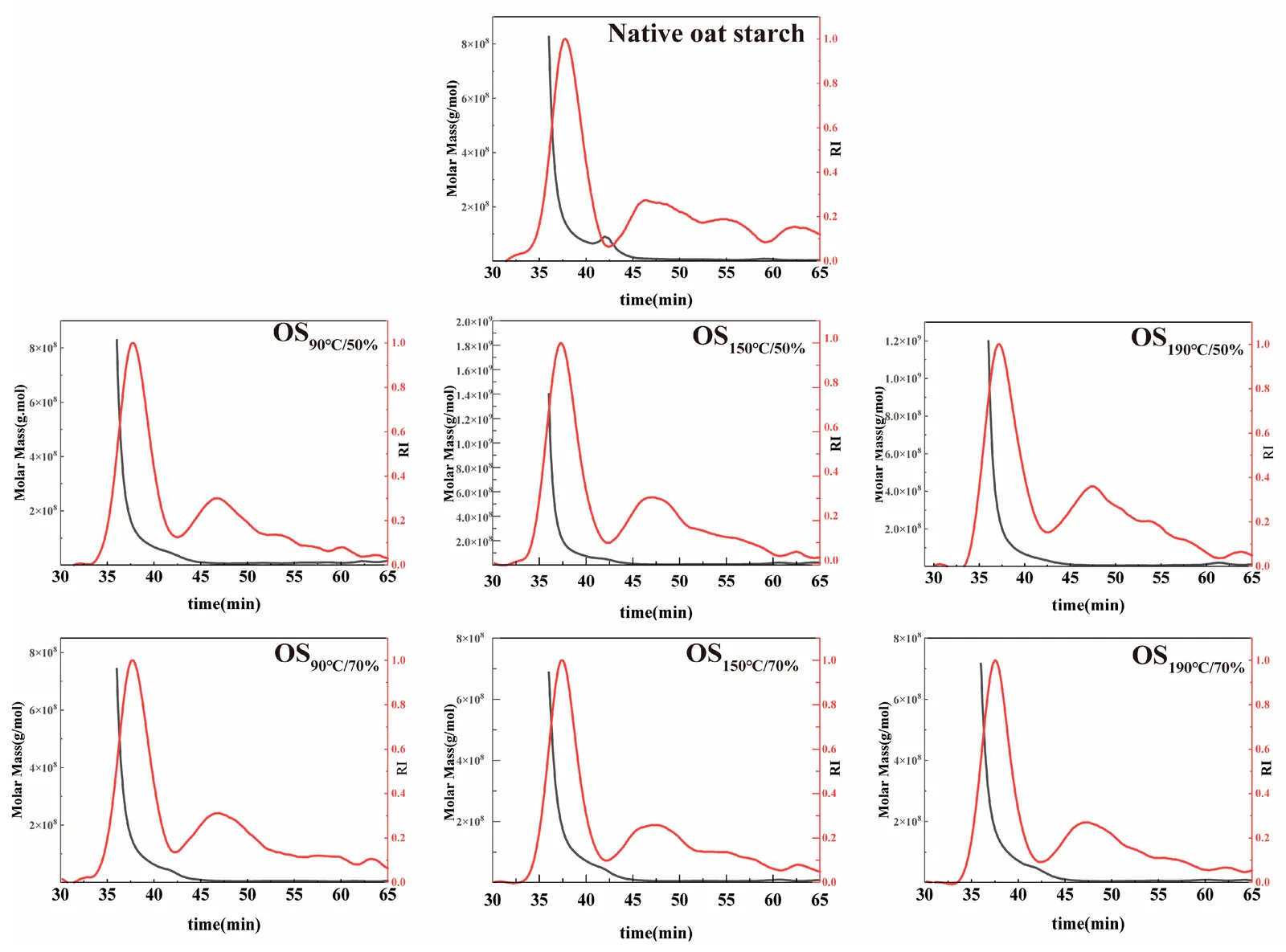
Size-exclusion chromatography–multi-angle laser light scattering–refractive index (SEC-MALLS-RI) chromatograms of native and subcritical ethanol–water-treated oat starch. Black curves: molar mass (left axis, g/mol); red curves: differential refractive index (RI) signal (right axis), plotted versus retention time (min). The two RI peaks correspond to the amylopectin and amylose fractions.

The changes in molecular weight distribution of modified oat starches are shown in Figure 4, with corresponding molecular weight parameters summarized in Table 1. In the refractive index (RI) chromatograms, all samples exhibited two characteristic peaks corresponding to amylopectin and amylose fractions [27,28]. Compared with native oat starch, the number-average molecular weight (Mn) of the modified starches fluctuated within the same order of magnitude. Notably, the polydispersity index (PDI) increased from 7.13 for native oat starch to 8.87 for OS_150°C/50%_ and 9.70 for OS_190°C/50%_, indicating a broader molecular weight distribution. Importantly, this broadening was not accompanied by a uniform reduction in molecular size: for several high-temperature samples, the Mw, Mp and Mz values exceeded those of native oat starch (Table 1); for OS_150°C/50%_, for example, Mw increased from 88,407.25 to 140,842.31 kDa, Mp from 143,600.95 to 241,748.03 kDa, and Mz from 278,614.64 to 520,402.16 kDa. Because both increases and decreases in the individual molecular weight averages were observed, these data indicate increased molecular weight heterogeneity rather than a single dominant mechanism; chain degradation and molecular reassociation are both possible, non-exclusive explanations.

### 3.7. Cold Water Swelling and Solubility

The cold-water solubility and swelling properties of modified oat starch are shown in Table 2. Swelling power reflects the ability of starch granules to bind to water, and solubility signifies the dissolution of starch molecules in water at a certain temperature [29].

Two-way ANOVA revealed significant main effects of temperature (F (5,12) = 2177.7, *p* < 0.001 swelling; F (5,12) = 1294.4, *p* < 0.001 solubility) and ethanol concentration (F (1,12) = 720.9, *p* < 0.001; F (1,12) = 1111.9, *p* < 0.001), and a significant temperature × ethanol interaction (F (5,12) = 107.2, *p* < 0.001; F (5,12) = 239.8, *p* < 0.001). As the modification temperature increased from 90 to 190 °C, both the cold-water solubility and swelling properties of oat starch increased. The cold-water swelling ability of oat starch increased from 2.13 g/g to 11.60 g/g (*p* < 0.05), the cold-water solubility increased from 1.05% to 13.76% (*p* < 0.05). With the ethanol concentration increases from 50% to 70%, the cold-water swelling ability of oat starch significantly decreased from 11.60 g/g to 7.65 g/g (*p* < 0.05), and the cold-water solubility decreased from 13.76% to 6.40% (*p* < 0.05). The cold-water swelling ability of modified oat starch decreased significantly with the increase in ethanol concentration. At 50% ethanol, swelling power increased continuously from 2.13 g/g (90 °C) to 11.60 g/g (190 °C). At 70% ethanol, swelling increased to a maximum of 8.03 g/g at 170 °C and then decreased to 7.65 g/g at 190 °C, indicating a non-monotonic trend at the higher ethanol concentration.

These results indicate that the modification temperature and ethanol concentration significantly affect the cold-water solubility and swelling of oat starch. The modification temperature caused changes in the microstructure of oat starch. As the temperature rises, the starch granules undergo significant swelling, forming a porous honeycomb structure and developing surface cracks. The microstructure of the starch granules possesses a larger surface area, which enhances the ability of water molecules to bind and adsorb onto the surface [30]. Therefore, higher modification temperatures promote cold-water solubility and swelling.

### 3.8. Gel-Forming Characteristics

**Figure 5:**
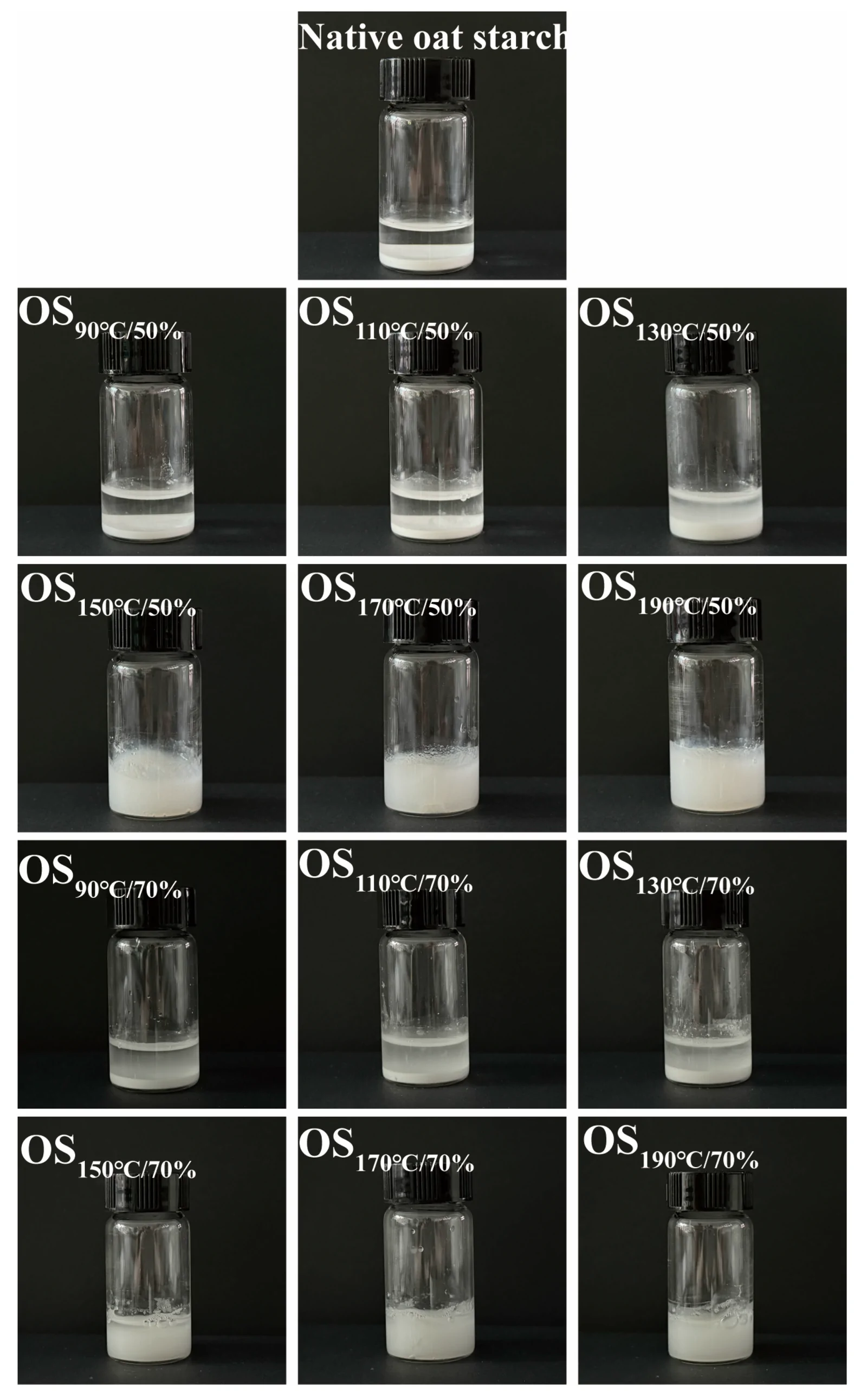
Photographs of cold-water gels formed by native and subcritical ethanol–water-treated oat starch (0.5 g starch in 4.5 g water, photographed after standing for 10 min), as a preliminary visual assessment of gelation.

The gel state of modified oat starch at room temperature was shown in Figure 5, as a preliminary visual assessment. A gel was recorded as “uniform” when no visible phase separation was observed, “stable” when no syneresis (water release) occurred within the 10 min observation, and “self-supporting” when it did not flow upon inversion of the vial. Native oat starch is insoluble in water and undergoes layer separation upon standing. At an ethanol concentration of 50%, the modified starch samples OS_90°C/50%_, OS_110°C/50%_, and OS_130°C/50%_ formed only a small amount of paste and exhibited pronounced separation, whereas OS_150°C/50%_ and OS_170°C/50%_ formed visually uniform, self-supporting gels. The gel of OS_190°C/50%_ was visually coarse. When the ethanol concentration was increased to 70%, a similar gelation trend was observed, but with improved fluidity: OS_150°C/70%_, OS_170°C/70%_, and OS_190°C/70%_ all formed visually homogeneous gels with a certain degree of fluidity. As shown in Table 2, the cold-water swelling power and solubility followed the same order: OS_190°C/50%_ > OS_170°C/50%_ > OS_150°C/50%_ > lower-temperature samples [31]. Because these observations are qualitative, objective rheological characterization (e.g., storage modulus G′ and loss modulus G″) should be performed in future work to confirm gel strength.

## 4. Discussion

This study provides the first systematic comparison of the formation of a new orthorhombic amylose phase in granular cold-water-swelling starch across a wide temperature range (90–190 °C) under subcritical ethanol–water conditions. The results indicate that high temperature promotes the solvation of the native nanocrystals and the crystallization of a new phase, while ethanol concentration modulates the morphological integrity of the granules during this process.

### 4.1. Crystalline Phases Formed at Low and High Temperatures

The XRD and DSC data collectively demonstrate a clear temperature threshold for the crystallization of the new orthorhombic phase. At ≤130 °C, the native A-type diffraction pattern persisted and the gelatinization endotherm remained detectable, indicating the retention of native crystalline domains and their associated thermal phase-transition behavior. Between 130 and 150 °C, the two crystalline phases coexist, which is consistent with a two-phase mixture rather than with an intermediate state of one transforming lattice. At ≥150 °C, the reflections of the new phase dominated and the gelatinization endotherm disappeared (50% ethanol), indicating that the native nanocrystals had been solvated and replaced by single-helical amylose. At 90–110 °C, the decrease in DSC residual enthalpy corresponded well with the reduction in XRD relative crystallinity, confirming the disruption of intrinsic crystalline order during partial gelatinization. Above 150 °C, no endotherm was detectable while the XRD crystallinity rebounded across 150–190 °C. This discrepancy suggests that the native crystalline structure was destroyed and replaced by the newly formed orthorhombic phase. The absence of a detectable endotherm could be attributed either to its high thermal stability within the investigated temperature window or to its rapid solvation and dissociation upon excess hydration prior to thermal scanning. This temperature-dependent crystallization is consistent with the mechanism proposed by Li et al., in which thermal energy is thought to drive amylose leaching and subsequent complex with ethanol, which likely acts as the helix-templating agent [32]; this mechanism is an interpretation based on our data and the literature, rather than a directly demonstrated mechanism. Higher ethanol concentration delayed the loss of birefringent order (the Maltese cross disappeared at 110 °C in 50% ethanol but only at 130 °C in 70% ethanol) but did not shift the temperature at which the gelatinization endotherm vanished, which remained 150 °C for both ethanol concentrations. Ethanol is likely the helix-templating agent, as suggested by our IR and XRD results, consistent with previous reports, thereby raising the energy barrier for complete structural disordering [33]. Although the OS_190°C/70%_ sample retained its granular morphology (Figure 2) and XRD crystallinity (Figure 3B), the absence of a gelatinization endotherm (Figure 3F) strongly suggests that the native orthorhombic nanocrystals were solvated and replaced by single-helical amylose, rather than simply amorphized.

### 4.2. Direct and Indirect Effects of Modification Temperature

It is important to distinguish the direct effect of temperature from its indirect effect through promoting starch gelatinization. At modification temperatures (90–130 °C), starch granules retain their orthorhombic crystal structure, and the limited granular swelling restricts amylose leaching into the aqueous–ethanol phase. Consequently, ethanol molecules have insufficient access to free amylose chains, thereby hindering the formation of single-helix inclusion complexes. At modification temperatures ≥ 150 °C, which is above the gelatinization threshold, the granule undergoes irreversible swelling and disruption, releasing amylose into the continuous phase where it can complex with ethanol. Ethanol interacts with the single-helical amylose chains and stabilizes their packing, driving the nucleation and growth of the new orthorhombic phase. Because ethanol is largely removed during drying, the resulting structure is single-helical amylose with empty cavities rather than an inclusion complexes. Therefore, the apparent promotion of the new orthorhombic phase by high temperature is mediated primarily through the degree of gelatinization rather than through a direct catalytic effect of temperature on the crystallization process itself. This mechanistic distinction is supported by the observation that at 70% ethanol concentration—where gelatinization is partially suppressed—the formation of the new phase is delayed relative to the 50% ethanol condition, despite both being subjected to the same thermal treatment.

### 4.3. Proposed Formation Mechanism of the New Orthorhombic Phase

Formation mechanism of the new orthorhombic phase. Two features of the data indicate that the new phase forms by crystallization and not by a transformation of the native phase. First, a transformation within a single lattice would change only the intensities and the number of allowed reflections. Here, a different set of reflections appears, at 2θ ≈ 8.0°, 13.9° and 21.2°, that cannot be indexed on the unit cell of the native nanocrystals, while the reflections of the native phase decrease and disappear. Second, the relative crystallinity increases from 33.77 ± 0.95% in native oat starch to 62.63 ± 3.49% in the samples treated at 150–190 °C, and this increase coincides with the disappearance of the gelatinization endotherm. A transition between two structures of the same crystalline entity cannot create this much additional crystalline order while the original nanocrystals lose their thermal signature. Solvation of the native nanocrystals followed by crystallization of a new phase can.

We therefore propose the following mechanism, in which the hydroxyl groups of ethanol act as the structuring agent and temperature acts mainly as the means by which amylose is released. In the first step, at 150 °C and above, the ethanol–water mixture penetrates the granule, the native orthorhombic nanocrystals are solvated, and their double helices are released as disordered amylose chains into the liquid phase. In the second step, ethanol competes with water for hydrogen bonding, lowers the water activity and the dielectric constant of the medium, and reduces the hydration shell of the amylose chain. Under these conditions, the chain adopts the left-handed single helix of six glucose residues per turn that is stabilized by intramolecular hydrogen bonds, and the hydroxyl group of ethanol hydrogen-bonds to the chain and templates this conformation. Water alone is a poor helix-forming partner, because it hydrates the chain rather than ordering it. In the third step, the helices associate side by side through van der Waals contacts and intermolecular hydrogen bonds, and pack into the orthorhombic arrangement that produces the reflections listed in Table S2. Cooling and washing with anhydrous ethanol act as an antisolvent step that fixes this packing and removes the guest, so that the product is single-helical amylose with empty cavities rather than an inclusion complex.

This mechanism accounts for three observations. It explains why the samples treated at 90–130 °C, in which the native nanocrystals survive, contain no new phase. It explains why 70% ethanol, which suppresses gelatinization and therefore limits the amount of amylose released into solution, gives weaker reflections of the new phase than 50% ethanol at the same temperature. It also explains why the new phase forms at 150 °C in this system, whereas gelatinization of starch in pure water at the same temperature yields an amorphous gel rather than a crystalline phase. The mechanism is a hypothesis derived from our X-ray diffraction, calorimetric and spectroscopic data and from the literature, and it is presented as a testable model rather than as a demonstrated pathway.

### 4.4. Morphological and Molecular Changes

The morphological evolution observed by SEM provides structural context for the functional property changes. At low temperatures (90–130 °C), granules remained largely intact with smooth surfaces, consistent with limited water penetration. At 150 °C in 50% ethanol, a distinctive porous honeycomb architecture emerged—a morphology previously associated with enhanced water uptake in ethanol-treated starches [34]. This structure likely arises from controlled swelling during the subcritical ethanol–water treatment, in which ethanol stabilizes the expanded framework; after cooling and filtration with anhydrous ethanol, both the residual water and ethanol were subsequently removed during drying, leaving the empty single-helical structure. At 170–190 °C, excessive thermal energy caused rupture, surface cracking, and agglomeration, indicating that structural integrity is preserved only within an optimal temperature window. By contrast, granules prepared at 70% ethanol retained their expanded but intact morphology even after high-temperature treatment, suggesting that higher ethanol concentrations may suppress gelatinization through hydrogen bonding with water [35,36]. The broad molecular weight distribution (PDI 5.80–9.70) observed by GPC indicates increased molecular weight heterogeneity after high-temperature processing. Because the Mw, Mp and Mz values of several high-temperature samples exceeded those of native starch while Mn remained comparable, chain degradation and intermolecular reassociation are both plausible and non-exclusive.

### 4.5. Relationship Between Structure and Cold-Water Functionality

The functional consequences of these structural transformations are manifested in cold-water swelling, solubility, and gelation. Swelling capacity increased approximately six-fold (from 2.13 to 11.60 g/g) as preparation temperature rose from 90 to 190 °C at 50% ethanol, corresponding to the development of the porous morphology and increased surface area for water binding. However, gel quality exhibited a non-monotonic relationship with temperature: uniform, stable gels formed at 150–170 °C, whereas gels prepared at 190 °C were qualitatively coarse, suggesting that excessive structural degradation compromises gel network homogeneity. At 70% ethanol, gel fluidity was improved across the high-temperature range, consistent with the more intact granular morphology. This trade-off between swelling capacity and gel quality highlights the need to balance structural disruption against granular integrity when designing GCWS starches for specific applications.

### 4.6. Comparison with Previous Studies

In relation to prior work, Dries et al. [10] reported the formation of the single-helical amylose phase at 149–182 °C under high-pressure conditions, and Zhang et al. [11] reported GCWS starch formation at 95 °C under atmospheric pressure. In the present study the reflections of the new orthorhombic phase were negligible at 90–130 °C, appeared at 150 °C, and increased in area with temperature. Low-temperature preparation therefore preserved finer granular morphology but yielded little of the new phase and modest swelling, whereas high-temperature preparation maximized the content of the new phase and cold-water functionality at the cost of partial granular disintegration. This trade-off has not been explicitly characterized in previous studies.

### 4.7. Limitations

Several limitations should be considered. Because no water-only heat-treated control was included, a purely thermal contribution to the structural changes cannot be excluded, and the mechanism proposed above is therefore presented as a hypothesis rather than as a demonstrated pathway. A water-only control, time-resolved diffraction during heating and cooling, and a wider ethanol concentration series are required to test it. The relationship between amylose chain-length distribution and the crystallization efficiency of the new phase was not directly probed. The scalability and energy cost of subcritical fluid processing at industrial scale also remain to be evaluated. Additionally, the intrinsic nanocrystals in native oat starch have an orthorhombic crystal structure. After subcritical ethanol–water treatment, a new, non-complex crystalline phase appears, whose reflections are consistent with a V6a-type orthorhombic unit cell (a ≈ 13.0 Å, b ≈ 23.0 Å, c ≈ 8.05 Å). This new phase is presumably formed by the interaction of amylose with ethanol, and its precise structure (exact space group and solvent occupancy) remains to be fully established.

## 5. Conclusions

This study examined how modification temperature and ethanol concentration govern the crystallization of a new phase and the physicochemical properties of oat starch across 90–190 °C. At 90–130 °C (low temperature), the nanocrystals with the native orthorhombic crystal structure were largely retained, whereas at 150–190 °C (high temperature) the new orthorhombic ethanol-induced amylose phase (OEA phase) accounted for most of the crystalline fraction. As the preparation temperature increased, cold-water swelling capacity rose from 2.13 to 11.60 g/g and solubility from 1.05% to 13.76%. However, while high temperatures enhanced swelling and solubility, granular integrity was compromised, indicating structural trade-offs. These results underscore the pivotal role of high temperature in facilitating the crystallization of the new orthorhombic phase, supporting potential applications in encapsulation, texture modification, and functional foods.

## Figures and Tables

**Table 1:** Molecular Weight Distribution of native and modified oat starch.

Samples	Mn (kDa)	Mw (kDa)	Mp (kDa)	Mz (kDa)	Polydispersity (Mw/Mn)
Native oat starch	12,399.78	88,407.25	143,600.95	278,614.64	7.13
OS_90°C/50%_	16,997.71	98,628.29	143,288.07	291,384.12	5.80
OS_150°C/50%_	15,882.07	140,842.31	241,748.03	520,402.16	8.87
OS_190°C/50%_	12,575.82	122,015.04	274,702.17	471,614.04	9.70
OS_90°C/70%_	11,655.74	86,633.57	148,709.63	273,401.34	7.43
OS_150°C/70%_	12,787.39	103,593.73	187,004.26	293,487.12	8.10
OS_190°C/70%_	13,122.57	104,069.14	172,547.52	288,919.21	7.93

Mn means Number Average Molecular Weight, Mw means Weight Average Molecular Weight, Mp means Peak Molecular Weight, Mz means Z-Average Molecular Weight. Molecular weights are absolute values determined by SEC-MALLS-RI (dn/dc = 0.07 mL/g), not calibrated values.

**Table 2:** Cold-water swelling and solubility properties of modified oat starch.

Samples	Cold-Water Swelling (g/g)	Cold-Water Solubility (%)
Native oat starch	1.99 ± 0.04	0.58 ± 0.22
OS_90°C/50%_	2.13 ± 0.00 ^e^	1.05 ± 0.16 ^g^
OS_110°C/50%_	2.21 ± 0.08 ^e^	1.21 ± 0.22 ^g^
OS_130°C/50%_	4.23 ± 0.06 ^d^	4.96 ± 0.07 ^d^
OS_150°C/50%_	9.70 ± 0.06 ^b^	6.56 ± 0.05 ^c^
OS_170°C/50%_	11.06 ± 0.09 ^a^	10.55 ± 0.41 ^b^
OS_190°C/50%_	11.60 ± 0.50 ^a^	13.76 ± 0.11 ^a^
OS_90°C/70%_	2.25 ± 0.04 ^e^	0.96 ± 0.15 ^g^
OS_110°C/70%_	2.28 ± 0.08 ^e^	2.08 ± 0.08 ^f^
OS_130°C/70%_	2.68 ± 0.08 ^e^	2.82 ± 0.14 ^f^
OS_150°C/70%_	7.65 ± 0.01 ^c^	4.14 ± 0.02 ^e^
OS_170°C/70%_	8.03 ± 0.10 ^c^	5.51 ± 0.35 ^d^
OS_190°C/70%_	7.65 ± 0.01 ^c^	6.40 ± 0.18 ^c^

Values are mean ± SD of *n* = 2 independently prepared batches. Different lowercase letters in the same column indicate significant differences (*p* < 0.05) among the twelve modified samples (both ethanol concentrations and temperatures, determined by two-way ANOVA (temperature × ethanol concentration) followed by Tukey’s HSD test. Native oat starch was treated as the prespecified control and was not included in the factorial analysis.

## Data Availability

The original contributions presented in this study are included in the article/Supplementary Materials. Further inquiries can be directed to the corresponding author.

## Supplementary Materials

The following supporting information can be downloaded at: https://www.mdpi.com/article/10.3390/foods15193506/s1, Figure S1: Microscopic morphology of native oat starch and modified oat starch; Figure S2: SEM images of native oat starch and modified oat starch. Table S1. Processing conditions and estimated autogenous pressure of each subcritical ethanol–water-treated oat starch sample. Table S2. Peak list and crystal-structure assignment for the subcritical ethanol–water treated oat starch (representative sample OS_150°C/50%_).

## References

[1] Rosales M.R., Luna H.E.R., Lozano D.C., Andrade-González I., Luna-Solano G. (2025). Functionality and Modifications of Food Hydrocolloids: An Approach to Starch. Starch-Stärke.

[2] Zhang J., Zhang M., Wang C., Zhang Y., A R., Bai X., Zhang Y., Zhang J. (2021). Effects of high hydrostatic pressure on microstructure, physicochemical properties and in vitro digestibility of oat starch/β-glucan mixtures. Int. J. Food Sci. Technol..

[3] Li Y., Qi Y., Li H., Chen Z., Xu B. (2022). Improving the cold water swelling properties of oat starch by subcritical ethanol-water treatment. Int. J. Biol. Macromol..

[4] He R., Li S., Zhao G., Zhai L., Qin P., Yang L. (2023). Starch Modification with Molecular Transformation, Physicochemical Characteristics, and Industrial Usability: A State-of-the-Art Review. Polymers.

[5] Jin Z.Y., Li J.X., Zhou X. (2021). Research Progress on Physical Preparation and Application of Cold-Water-Soluble Starch. J. Food Sci. Technol..

[6] Lee H., Yoo B. (2023). Particle Agglomeration and Properties of Pregelatinized Potato Starch Powder. Gels.

[7] Cao J., Liu W., Zhang T., Zhang L. (2018). Preparation and characterization of eucalyptus wood flour/polypropylene composites by subcritical ethanol and 3-Aminopropyltriethoxysilane as cosolvent. Polym. Compos..

[8] Wu Y., Yang R., Zhu W., Wan J. (2026). Preparation of V-type granular starch in natural deep eutectic solvent and its physicochemical properties and in vitro digestibility. Int. J. Biol. Macromol..

[9] Jin Z. (2018). Functional Starch and Applications in Food.

[10] Dries D.M., Gomand S.V., Delcour J.A., Goderis B. (2016). V-type crystal formation in starch by aqueous ethanol treatment: The effect of amylose degree of polymerization. Food Hydrocoll..

[11] Zhang B., Dhital S., Haque E., Gidley M.J. (2012). Preparation and characterization of gelatinized granular starches from aqueous ethanol treatments. Carbohydr. Polym..

[12] He Y.P., Han X.X., Zhao Y.J., Hao C.M., Zheng X.L. (2011). Comparison of separation oat starch by alkali method and ultrasound-assisted enzymatic method. Food Mach..

[13] Guo C., Han F., Geng S., Shi Y., Ma H., Liu B. (2023). The physicochemical properties and Pickering emulsifying capacity of acorn starch. Int. J. Biol. Macromol..

[14] Zhan Q., Ye X., Zhang Y., Kong X., Bao J., Corke H., Sui Z. (2020). Starch granule-associated proteins affect the physicochemical properties of rice starch. Food Hydrocoll..

[15] Li M.-Y., An F.-K., Luo H.-L., Liu Y., Fu Z. (2023). Physicochemical properties and digestion characteristics of acylated *Arenga pinnata* starches. Int. J. Food Sci. Technol..

[16] Syahariza Z.A., Li E., Hasjim J. (2010). Extraction and dissolution of starch from rice and sorghum grains for accurate structural analysis. Carbohydr. Polym..

[17] Lin L., Guo D., Zhao L., Zhang X., Wang J., Zhang F., Wei C. (2016). Comparative structure of starches from high-amylose maize inbred lines and their hybrids. Food Hydrocoll..

[18] Li E., Hasjim J., Dhital S., Godwin I.D., Gilbert R.G. (2011). Effect of a gibberellin-biosynthesis inhibitor treatment on the physicochemical properties of sorghum starch. J. Cereal Sci..

[19] Gani A., Ashwar B.A., Akhter G., Shah A., Wani I.A., Masoodi F.A. (2017). Physico-chemical, structural, pasting and thermal properties of starches of fourteen Himalayan rice cultivars. Int. J. Biol. Macromol..

[20] Zhang M., Chen G., Li M., Niu H., Chen Y., Jiang P., Li S. (2022). Effects of microwave on microscopic, hydration, and gelatinization properties of oat and its application on noodle processing. Food Process. Preserv..

[21] Rodriguez-Garcia M.E., Hernandez-Landaverde M.A., Delgado J.M., Ramirez-Gutierrez C.F., Ramirez-Cardona M., Millan-Malo B.M., Londoño-Restrepo S.M. (2021). Crystalline Structures of the Main Components of Starch. Curr. Opin. Food Sci..

[22] Shi L., Hopfer H., Ziegler G.R., Kong L. (2019). Starch-menthol inclusion complex: Structure and release kinetics. Food Hydrocoll..

[23] Xu M., Zhao X., Chen Z., Zhou Z., Ji S., Xu Y., Zhang C., Shen J., Chen Q., Li K. (2025). Insights into the improved cold-water solubility and digestibility of alkaline-alcohol modified cassava starch: A discussion from the perspective of fine structure. Int. J. Biol. Macromol..

[24] Zhao H., Zhang H., Xu Q., Zhang H., Yang Y. (2023). Thermal, Rheological, Structural and Adhesive Properties of Wheat Starch Gels with Different Potassium Alum Contents. Molecules.

[25] Lopez-Leon A., Munoz M.E.P., Luna P.G.E., Millan-Malo B., Bello-Pérez L.A., Hoyos-Leyva J.D., Rodriguez-Garcia M.E. (2026). Retrogradation in rice starch: Nanocrystals from orthorhombic to hexagonal crystal structure crystallization. Int. J. Biol. Macromol..

[26] Martinez-Munoz P.E., Cortez G.E., Urbiola H.I.M., Lopez-Leon A., Barron-Garcia O.Y., Rodriguez-Garcia M.E. (2025). Physicochemical characterization of isolated nanocrystals in starch and isolated rice starch: A new perspective. Food Chem..

[27] Li B., Wang S., Zhang Y., Huang C., Zhao Y., Wu G., Tan L. (2023). Effect of the Amylose Nanoscale Polymerization Index on the Digestion Kinetics and Mechanism of Recombinant Chinese Seedless Breadfruit Starch Triadic Complexes. J. Agric. Food Chem..

[28] Cheetham N.W.H., Tao L. (1997). The effects of amylose content on the molecular size of amylose, and on the distribution of amylopectin chain length in maize starches. Carbohydr. Polym..

[29] Li Y., Liang C., Liu J., Zhou C., Wu Z., Guo S., Liu J., A N., Wang S., Xin G. (2023). Moderate Reduction in Nitrogen Fertilizer Results in Improved Rice Quality by Affecting Starch Properties without Causing Yield Loss. Foods.

[30] Qi M., Jiang L., Song J., Han F., Xu M., Li Y., Ma C., Chen S., Li H. (2024). Investigating the Impact of Moisture Levels on Structural Alterations and Physicochemical Properties of Cassava Flour through Extrusion: A Comprehensive Study. Plant Foods Hum. Nutr..

[31] Zhao S.-C., Wang H., Kang H., Kong B.-H., Hu G.-S., Liu Q. (2019). Physicochemical and functional properties of barley starch from different varieties. Food Res. Dev..

[32] Li J., Zhou X., Jin Z. (2021). Effect of high-temperatures and aqueous ethanol treatment on the formation process and properties of V-type Granular Starch (VGS). Carbohydr. Polym..

[33] Zhang B., Dhital S., Flanagan B.M., Gidley M.J. (2014). Mechanism for starch granule ghost formation deduced from structural and enzyme digestion properties. J. Agric. Food Chem..

[34] Sarifudin A., Keeratiburana T., Soontaranon S., Tangsathitkulchai C., Tongta S. (2020). Pore characteristics and structural properties of ethanol-treated starch in relation to water absorption capacity. LWT.

[35] Li J.X., Capuano E., Tong L.T. (2025). Transformation of native starch into V-type granular starch through ethanol-aqueous heat treatment and its swelling behavior in cold water. Carbohydr. Polym..

[36] Majzoobi M., Farahnaky A. (2020). Granular cold-water swelling starch; properties, preparation and applications, a review. Food Hydrocoll..

