# Supplementary material for: Comparative Study on the Formation, Structure, and Properties of Granular Cold-Water-Swelling Oat Starch by Subcritical Ethanol–Water Treatment at High and Low Temperatures
Source: Foods. 2026 Oct 1;15(19):3506. doi: 10.3390/foods15193506 (PMC13665409; doi:10.3390/foods15193506)
Supplement: Supplementary file 1 [file foods-15-03506-s001.zip › foods-4451642-supplementary.pdf]

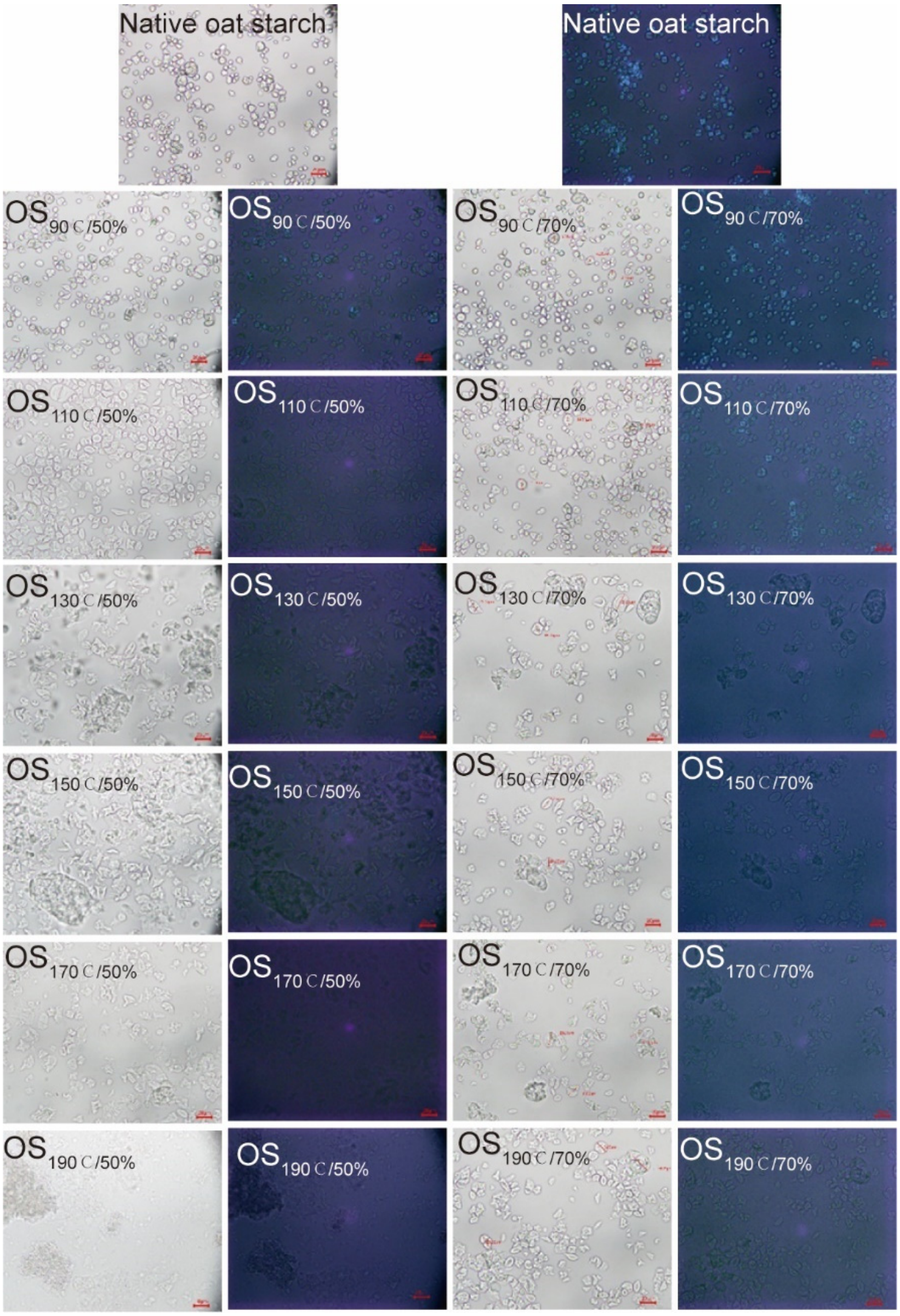

Figure S1. Microscopic morphology of native oat starch and modified oat starch.

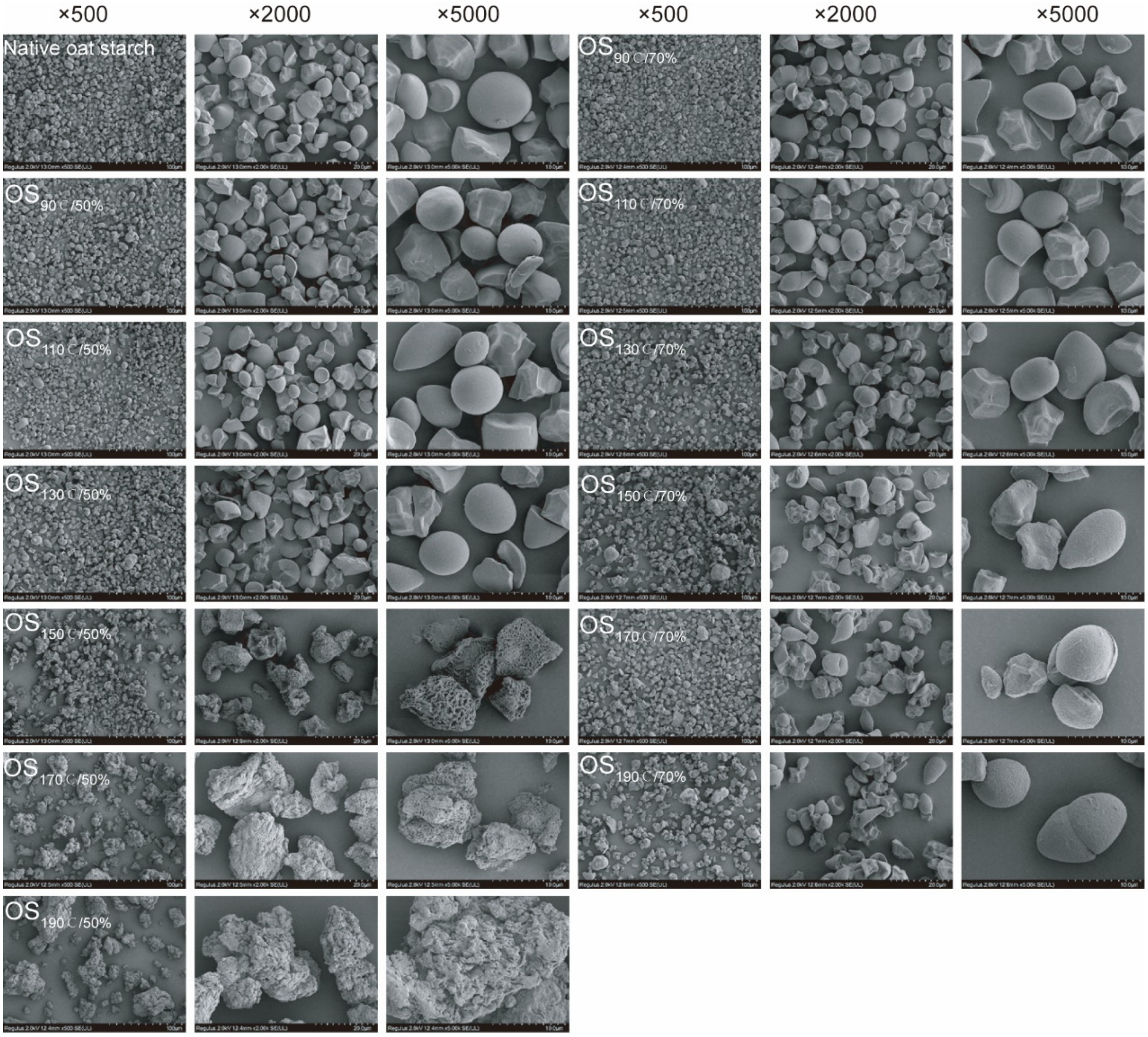

Figure S2. SEM images of native oat starch and modified oat starch.

Table S1. Processing conditions and estimated autogenous pressure of each subcritical ethanol–water-treated oat starch sample.

Sample	Ethanol conc. (% v/v)	Temperature (°C)	Holding time (min)	Autogenous pressure (MPa)*
$OS_{90°C/50\%}$	50	90	20	0.09
$OS_{110°C/50\%}$	50	110	20	0.19
$OS_{130°C/50\%}$	50	130	20	0.35
$OS_{150°C/50\%}$	50	150	20	0.61
$OS_{170°C/50\%}$	50	170	20	1.02
$OS_{190°C/50\%}$	50	190	20	1.61
$OS_{90°C/70\%}$	70	90	20	0.11
$OS_{110°C/70\%}$	70	110	20	0.22
$OS_{130°C/70\%}$	70	130	20	0.41
$OS_{150°C/70\%}$	70	150	20	0.71
$OS_{170°C/70\%}$	70	170	20	1.17
$OS_{190°C/70\%}$	70	190	20	1.85

*Autogenous pressures were estimated from the vapor pressure of the ethanol–water mixture using Raoult's law and the Antoine equation (ideal estimate; the real non-ideal mixture may develop somewhat higher pressure, plus ≈0.1 MPa from residual air in the headspace). All treatments were performed in a sealed high-temperature reactor (KH-50 mL) with a solid-to-liquid ratio of 1:9 (g/mL).

Table S2. Peak list and crystal-structure assignment for the subcritical ethanol–water treated oat starch (representative sample $OS_{150°C/50\%}$).

Peak	2θ (°)	d (Å)	Assignment	hkl*
1	8.0	11.04	New orthorhombic phase (OEA phase)	(110)
2	13.9	6.36	New orthorhombic phase (OEA phase)	(200)
3	15.4	5.75	Native orthorhombic nanocrystals	(200)
4	17.3	5.12	Native orthorhombic nanocrystals	(031)
5	18.3	4.84	Native orthorhombic nanocrystals	(220)
6	20.0	4.44	Native orthorhombic nanocrystals	(040)
7	21.2	4.19	New orthorhombic phase (OEA phase)	(310)
8	23.3	3.81	Native orthorhombic nanocrystals	(231)

d values were calculated from 2θ using the Bragg equation (Cu Kα1, λ = 1.5406 Å).

*hkl indices are preliminary and were checked against the A-type orthorhombic unit cell (a ≈ 11.7 Å, b ≈ 17.6 Å, c ≈ 10.7 Å) and the V6a-type orthorhombic unit cell of single-helical amylose (a ≈ 13.0 Å, b ≈ 23.0 Å, c ≈ 8.05 Å; Shi et al., 2019). All observed peaks are consistent with the orthorhombic crystal system (the native orthorhombic nanocrystals and the new orthorhombic phase).
